# Impact of simultaneous placement of implant and block bone graft substitute: an in vivo peri-implant defect model

**DOI:** 10.1186/s40824-021-00245-3

**Published:** 2021-11-25

**Authors:** Minh Khai Le Thieu, Amin Homayouni, Lena Ringsby Hæren, Hanna Tiainen, Anders Verket, Jan Eirik Ellingsen, Hans Jacob Rønold, Johan Caspar Wohlfahrt, Antonio Gonzalez Cantalapiedra, Fernando Maria Guzon Muñoz, Maria Permuy Mendaña, Ståle Petter Lyngstadaas, Håvard Jostein Haugen

**Affiliations:** 1 Department of Biomaterials, Institute of Clinical Dentistry, University of Oslo, 0317 Oslo, Norway; 2 Department of Periodontology, Institute of Clinical Dentistry, Faculty of Dentistry, University of Oslo, Oslo, Norway; 3 Department of Prosthetic Dentistry and Oral Function, Institute of Clinical Dentistry, Faculty of Dentistry, University of Oslo, Oslo, Norway; 4 Universidade de Santiago de Compostela, Facultad de Veterinaria, Campus Universitario, s/n, 27002 Lugo, Spain; 5 Ibonelab S.L., Laboratory of Biomaterials, Avda. da Coruña, 500 (CEI-NODUS), 27003 Lugo, Spain

**Keywords:** Bone substitute, Animal experimentation, Bone ring technique

## Abstract

**Background:**

Insufficient bone volume around an implant is a common obstacle when dental implant treatment is considered. Limited vertical or horizontal bone dimensions may lead to exposed implant threads following placement or a gap between the bone and implant. This is often addressed by bone augmentation procedures prior to or at the time of implant placement. This study evaluated bone healing when a synthetic TiO_2_ block scaffold was placed in circumferential peri-implant defects with buccal fenestrations.

**Methods:**

The mandibular premolars were extracted and the alveolar bone left to heal for 4 weeks prior to implant placement in six minipigs. Two cylindrical defects were created in each hemi-mandible and were subsequent to implant placement allocated to treatment with either TiO_2_ scaffold or sham in a split mouth design. After 12 weeks of healing time, the samples were harvested. Microcomputed tomography (MicroCT) was used to investigate defect fill and integrity of the block scaffold. Distances from implant to bone in vertical and horizontal directions, percentage of bone to implant contact and defect fill were analysed by histology.

**Results:**

MicroCT analysis demonstrated no differences between the groups for defect fill. Three of twelve scaffolds were partly fractured. At the buccal sites, histomorphometric analysis demonstrated higher bone fraction, higher percentage bone to implant contact and shorter distance from implant top to bone 0.5 mm lateral to implant surface in sham group as compared to the TiO_2_ group.

**Conclusions:**

This study demonstrated less bone formation with the use of TiO_2_ scaffold block in combination with implant placement in cylindrical defects with buccal bone fenestrations, as compared to sham sites.

## Background

Dental implants are commonly used to replace missing teeth. Implants were originally placed in healed alveolar ridges following physiologic bone resorption after tooth extraction [1]. With the change towards prosthetically-driven implant placement, the ideal implant position may present challenging bone architecture which may render exposure of implant threads or a gap between the alveolar bone and the implant. A peri implant gap of less than 2-2.25 mm often leads to spontaneous healing as demonstrated in several studies [2, 3]. For gaps exceeding 2 mm or exposed implant threads, the use of a graft material has been recommended, but there is a controversy as to which material is superior [4].

Autologous bone grafts are considered the gold standard for bone augmentation, but are associated with donor site morbidity and unpredictable graft resorption [5–7]. Allografts and xenografts are widely used and well documented [8], but run the risk of disease transmission from graft to host [9]. Synthetic bone graft substitutes circumvent many disadvantages with auto-, allo- and xenografts, which may lead to its increased use also reflected by recent regulatory changes by the European Union [10].

Clinical procedures combining vertical bone augmentation and implant placement are often performed in two stages. Currently, only few studies have reported on the use of block grafts in circumferential peri-implant defects in conjunction with implant placement, such as the bone ring technique [11, 12]. However the technique is scarcely reported in the literature, lacking control groups and only reporting short follow-up times [13]. Gaikwad et al. suggested that results from animal studies on autogenous bone rings were not sufficiently robust to support this technique in humans [14]. Previous studies on the bone ring technique are also widely heterogeneous with regard to graft materials, surgical protocols and peri-implant defect size and shape. As described by Botticelli et al., circumferential defects with a gap distance to the implant of up to 2.25 mm heal spontaneously, but if a concomitant buccal dehiscence was present, healing was incomplete [2].

This study evaluates a porous ceramic TiO_2_ bone graft substitute in a peri-implant defect model with a buccal dehiscence. TiO_2_ scaffolds have documented biocompatible properties with compressive strength similar to trabecular bone [15, 16]. The average pore size close to 400 μm, high porosity and interconnectivity gives a favourable pore architecture for osteogenesis [17–19]. Studies in minipigs have demonstrated bone ingrowth and angiogenesis within the structures of the TiO_2_ scaffolds in extraction sockets and in peri-implant dehiscence defects [18, 19].

In a buccal dehiscence model by Botticelli et al. particulate graft materials were assessed [20]. Using a synthetic block material may be more advantageous in such a model as targeted porosity and interconnectivity can be designed, which is impossible with the use of particulate grafts. Therefore, in the present study a TiO_2_ block scaffold was used simultaneous to implant placement for bone augmentation of 2.5 mm circumferential defects with buccal dehiscences in an experimental minipig model.

The aim of this experimental study was to evaluate the use of a TiO_2_ block scaffold in the bone ring technique for peri-implant bone defects with buccal fenestrations, without the use of barrier membranes, to evaluate bone healing.

## Methods

Ceramic TiO_2_ scaffolds were fabricated by polymer foam replication as described by Tiainen et al. [16]. TiO_2_ slurry was prepared by gradually dispersing 65 g of TiO_2_ powder (Kronos 1171, Kronos Titan GmbH, Leverkusen, Germany) cleaned with 1 M NaOH into 25 ml of sterilised H_2_O and stirred at 5000 rpm for 2.5 h. The slurry was adjusted to pH 1.5 during the stirring with 1 M HCl. Cylindrical polyurethane foam templates (60 ppi, Bulbren S, Eurofoam GmbH, Wiesbaden, Germany) were soaked in the slurry and squeezed to remove excess slurry. The polymer sponge was burned out before sintering at 1500 °C for 20 h. After sintering, the scaffolds were immersed with a slurry containing 40 g of powder dispersed in 25 ml of sterilised water. Excess slurry was removed by centrifugation and the second coating was sintered at 1500 °C for 20 h. The finished scaffolds were 7.0-7.5 mm in diameter and 5 mm in height. Prior to use, the scaffolds were steam sterilised at 121 °C for 20 min.

### Animals

Six female minipigs (Göttingen minipig, *Sus scrofa*, Ellegaard AS, Dalmose, Denmark) aged 27-32 months and weighing 42-51 kg were used. The animals were kept in a centre for large experimental animals at the Veterinary Teaching Hospital Rof Codina in Lugo, Spain. The animals were kept on a soft diet and subjected to oral hygiene by mechanical cleaning once every 3 weeks during the experimental study. The Regional Ethics Committee for Animal Research of the University of Santiago de Compostela approved the protocol (Ref. AE-LU-001/002/14).

All surgical procedures were performed under general anaesthesia and sterile conditions in an operating room using propofol (2 mg/kg/i.v., Propovet, Abbott Laboratories, Kent, UK) and 2.5–4% of isoflurane (Isoba-vet, Schering-Plough, Madrid Spain) for the entire period of the surgery. Lidocaine 2% with epinephrine 1:100.000 (2% Xylocaine Dental, Dentsply, York, PA, USA) was infiltrated locally to reduce intra-operatory bleeding and provide local analgesia. The animals were premedicated with acepromazine (0.05 mg/kg/i.m., Calmo Neosan, Pfizer, Madrid, Spain) and pain controlled with the administration of morphine (0.3 mg/kg/i.m., Morfina Braun 2%, B. Braun Medical, Barcelona, Spain). During anaesthesia, a veterinarian continuously monitored the animals.

### Experimental design

Mandibular premolars were extracted 4 weeks before implant placement. Mucoperiosteal flaps were bilaterally reflected and teeth carefully removed after tooth separation. Primary healing was accomplished by mattress sutures. Prophylactic administration of cefovecin sodium (8 mg/kg body weight S.C. S.I.D., Convenia®, Zoetis, Spain) was given postoperatively.

Four weeks after extraction, full thickness flaps were raised on the buccal and lingual side and the alveolar crest was levelled to create a flat surface for osteotomies. In each mandibular quadrant, two implant osteotomies were prepared. Thereafter, peri-implant defects were made with a trephine bur, 8 mm in diameter and 5 mm in height (Meisinger Bone Management® Systems, Hager & Meisinger GmbH, Neuss, Germany). The defects were placed with a minimum of 10 mm between the osteotomy centres, and at least 6 mm from neighbouring teeth. The defects were made so that the buccal portion of the defect wall was missing. Dental implants (OsseoSpeed TX 3.0 S 11 mm, Dentsply) were placed in the centre of the defect with the coronal 5 mm of the implants exposed.

**Fig. 1:**
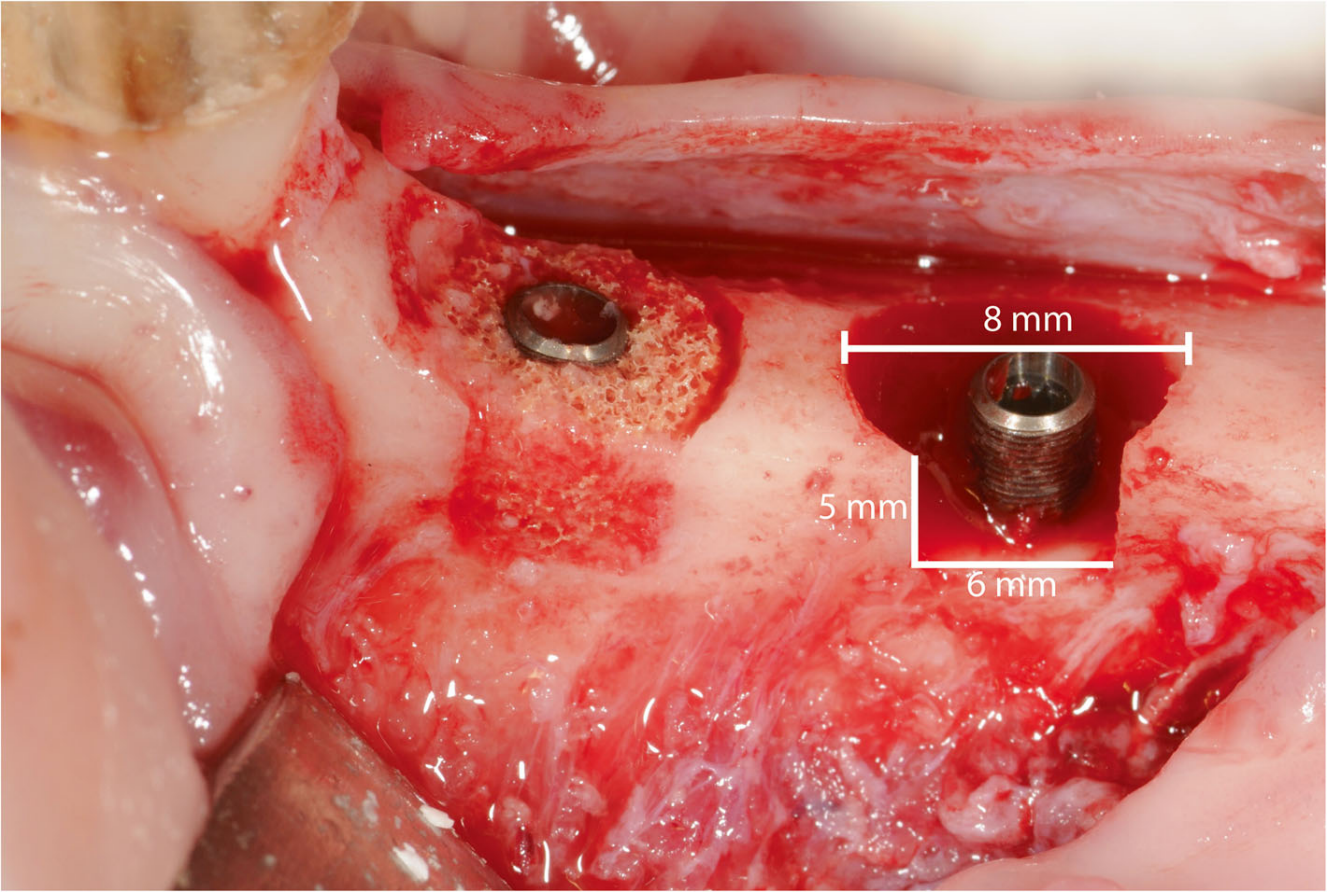
Clinical photos depicting the experimental model. Circumferential defects were created before implant placement with a block scaffold (TiO_2_ group) and implant only (sham)

The treatment allocation was assigned by a split mouth design where sham and TiO_2_ alternated between anterior and posterior location for every other animal. For the sites allocated to treatment with the TiO_2_ scaffold, a cylindrical block scaffold was perforated in the centre, creating a donut-shaped scaffold to fit around the implant and shaped to fit the defect with burs or scissors. The scaffold was secured in the defect by press-fit installation before the flap was passively adapted and primary wound closure was obtained (Fig. 1). After 12 weeks of healing, the animals were euthanized using a lethal dose of sodium Pentothal (40–60 mg/kg/i.v., Dolethal, Vetoquinol, France) and the mandibles were dissected and fixed in formalin.

### Micro-computed tomography scanning

Microcomputed tomographic imaging (microCT) of the samples was performed (SkyScan 1172, Bruker microCT, Kontich, Belgium) with a resolution of 7 μm, using 100 kV and 100 μA, with 3 average images every 0.4 degrees for a 360-degree rotation with an aluminium and copper filter. The data were reconstructed using NRecon software (Bruker microCT, Kontich, Belgium) with ring artefact correction of 10%.

**Fig. 2:**
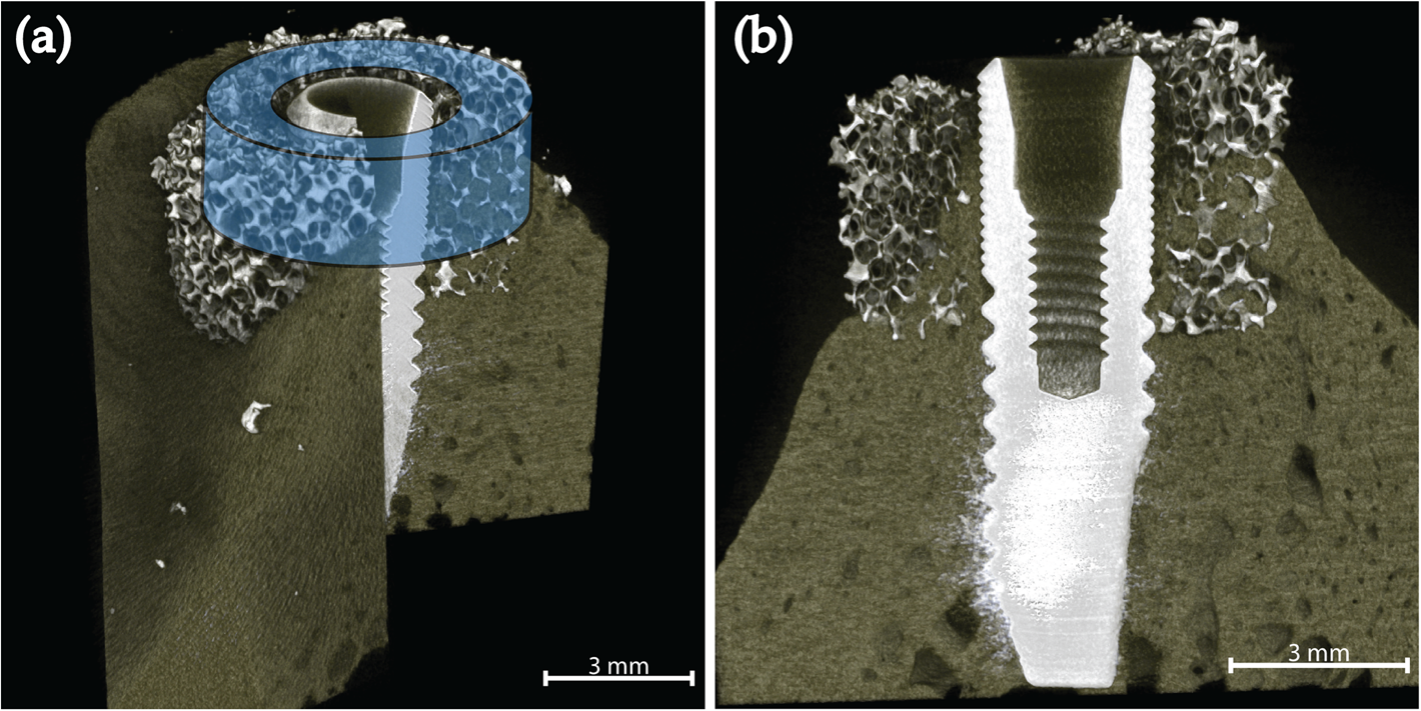
3D reconstructions of the microCT scan (**a**) Illustration of circumferential area evaluated for defect fill in blue. (**b**) Cross section of the same sample in bucco-lingual direction (buccal left)

The defect fill volume was analysed for a volume of interest (VOI) slightly smaller than the scaffold, to avoid possible errors at the interfaces of the scaffold’s outer and inner border (Fig. 2). The VOI was a cylindrical donut-shaped area with a height of approximately 2.3 mm (including the first ten upper threads of the implant) and a diameter of 6.0 mm concentric with the implant. Individual thresholds was set to measure the defect fill of bone and scaffold. Percentage of defect fill was analysed in CTan (Bruker microCT, Kontich, Belgium).

### Histological preparation

The tissue blocks were dehydrated in different graded ethanol series (70-100%), and infiltrated with four different graded mixtures of ethanol and infiltrating resin, glycometacrylate (Technovit 7200® VLC, Heraus Kulzer GMBH, Werheim, Germany). The samples were then polymerised, first under low intensity UV light for 4 h, followed by high intensity UV light for 12 h. Finally, the samples were placed in an oven at 37 °C for 24 h to assure a complete polymerisation. Longitudinal sections in bucco-lingual direction were obtained by cutting with a band saw and mechanically micropolished (Exakt Apparatebau, Norderstedt, Germany) with silicon carbide papers until a thickness for approximately 50 μm was obtained. The slides were stained with Levai Laczko for both histological examination and histomorphometric analysis.

All sections were observed using light microscopy and a PC-based image capture system (BX51, DP71, Olympus Corporation, Japan) and histometrically analysed. Quantitative histology was performed by a masked examiner using PC-based image analysis programs: Cell-sens 1.13 (Olympus Corporation, Japan) and Image Pro-Premier 9.0 (Media cybernetics, Bethesda, MD, USA).

### Histological analyses

**Fig. 3:**
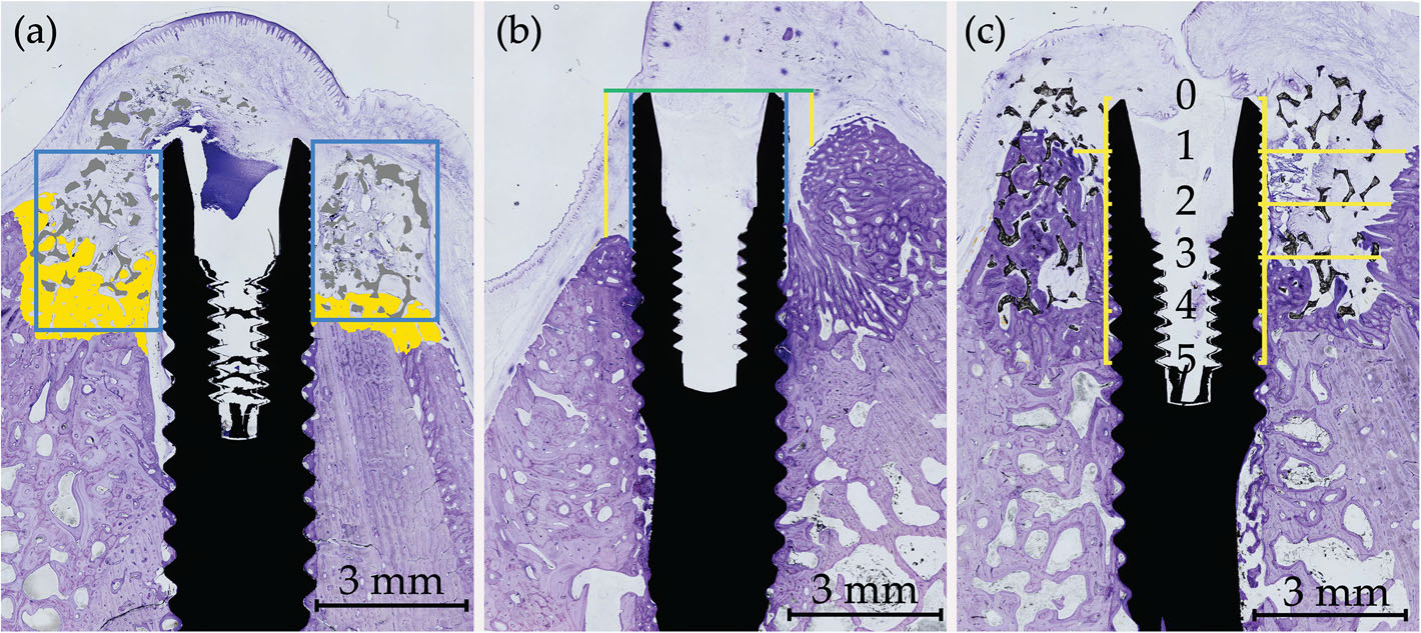
Histological samples (**a**) Illustration of defect analysis. Blue box depicting regions of interest in the buccal and lingual sides, yellow = bone, grey = TiO_2_ scaffold. **b** Vertical linear measurements from the implant top (green line) to the first bone contact at the implant surface (blue line) and 0.5 mm lateral to the implant (yellow line). **c** Horizontal linear measurements from implant surface to the first bone contact. Measured from implant top and five consecutive millimetres apically. Lingual side left and buccal side right for all figures

The images were painted with a digital tablet (Cintiq, Wacom, Japan) and Photoshop CS (Adobe, USA). The region of interest was the buccal and lingual rectangles defined from the top of the implant to the bottom of the defect created and from the surface of the implant to the lateral portion of the defect. The fraction of possible bone fill within each rectangle was calculated, excluding scaffold material in the TiO_2_ group (Fig. 3a).

Vertical linear measurements were made for two distances: 1) Implant shoulder to the first bone contact (first bone-to-implant contact, FBIC) and 2) Implant shoulder to the first bone contact 500 μm lateral to the implant (FBIC500) (Fig. 3b). Percentage of bone-to-implant contact (%BIC) was calculated for the first 5 mm from the implant top.

Horizontal bone growth within the defect was measured from the implant surface to the first bone contact in the buccal and lingual direction. Measurements were done in millimetre increments from the top of the implant to defect bottom 5 mm apically. The defect dimensions were set as reference points, hence when no bone was seen the value was set at a maximum 3 mm (Fig. 3c).

All measurements were done for both the buccal and lingual side of the implants using ImageJ.

(ImageJ 1.52a, National Institutes of Health, USA).

**Fig. 4:**
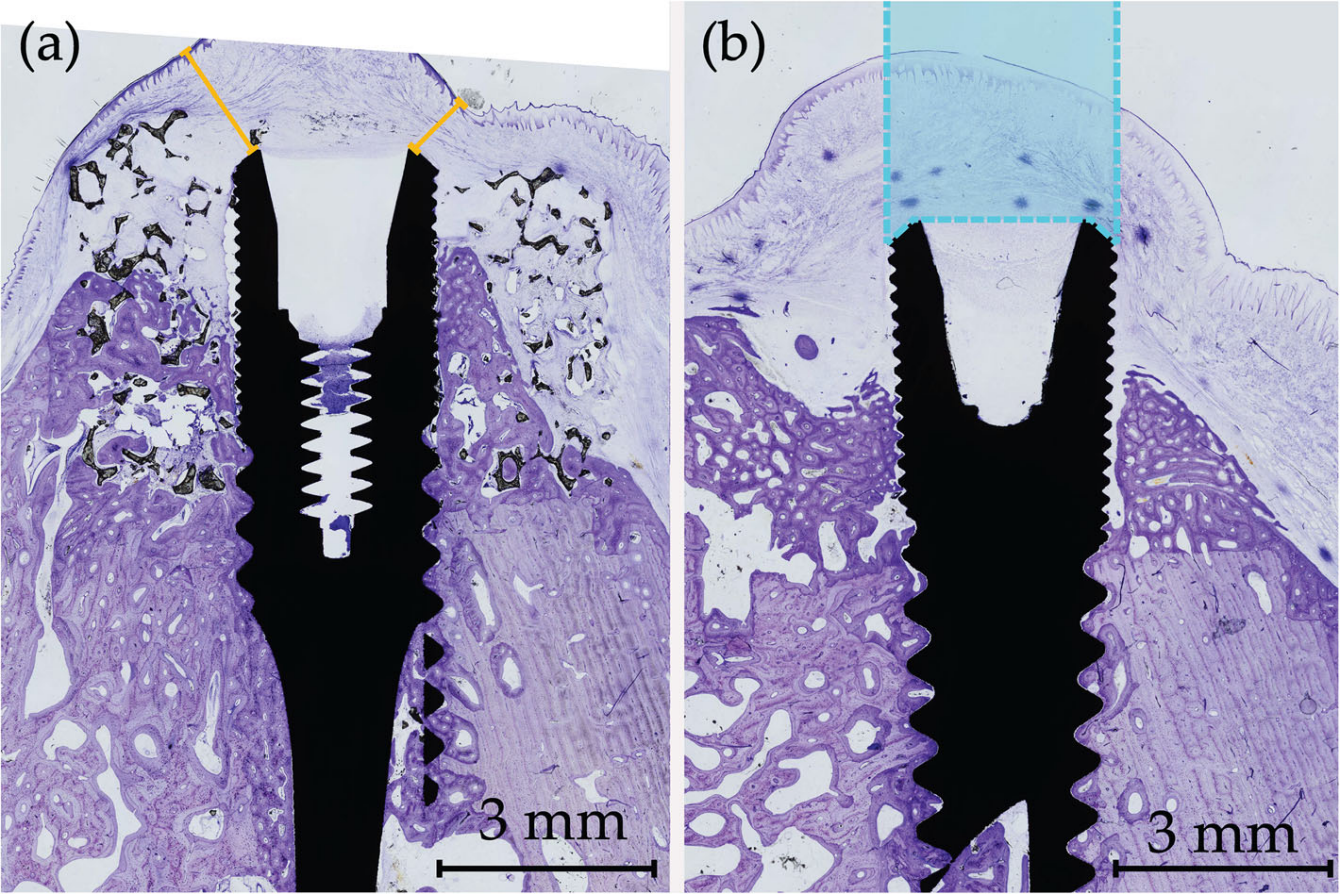
Soft tissue measurements in histological samples. Lingual side left and buccal side right for both figures. **a** Shortest distance from implant shoulder to oral cavity marked in yellow. **b** Area of soft tissue above the implant measured within the dotted blue lines

Linear measurements of soft tissue dimensions were performed for the shortest distance from implant shoulder to the oral cavity using ImageJ (Fig. 4a). The area of soft tissue above the implants was measured in Photoshop CS6 (Fig. 4b). One sample was excluded from the analysis due to missing soft tissue following the histological preparation.

### Statistics

Comparison across the two groups was performed using a paired t-test between contralaterals when normality was assumed and Wilcoxon Signed Rank Test when normality test failed. All statistical analyses were performed using SigmaPlot 14 (Systat Software, San Jose, CA, USA). Statistical significance was set at the 0.05 level.

## Results

Post-operative healing following surgery was uneventful. Five implants presented small mucosal perforations to the oral cavity at the time of harvest (Fig. 3c), four in the TiO_2_ scaffold sites and one in the sham sites.

### MicroCT

The apical portion of the implants placed in sound bone showed osseointegration at all sites, but the defect areas showed large variation of bone fill. The largest variations were seen in the TiO_2_ scaffold sites. One site was missing most of the scaffold and was subsequently excluded from further microCT analysis. Two sites demonstrated a fracture of approximately one fourth of the scaffold, one of which was missing while the other remained in the defect. The remaining nine scaffolds maintained their structural integrity. All but the excluded scaffold demonstrated bone growth within the porous scaffold structures in various degrees. As for the sham sites, three sites demonstrated almost complete bone coverage of the implants.

### Histology

**Fig. 5:**
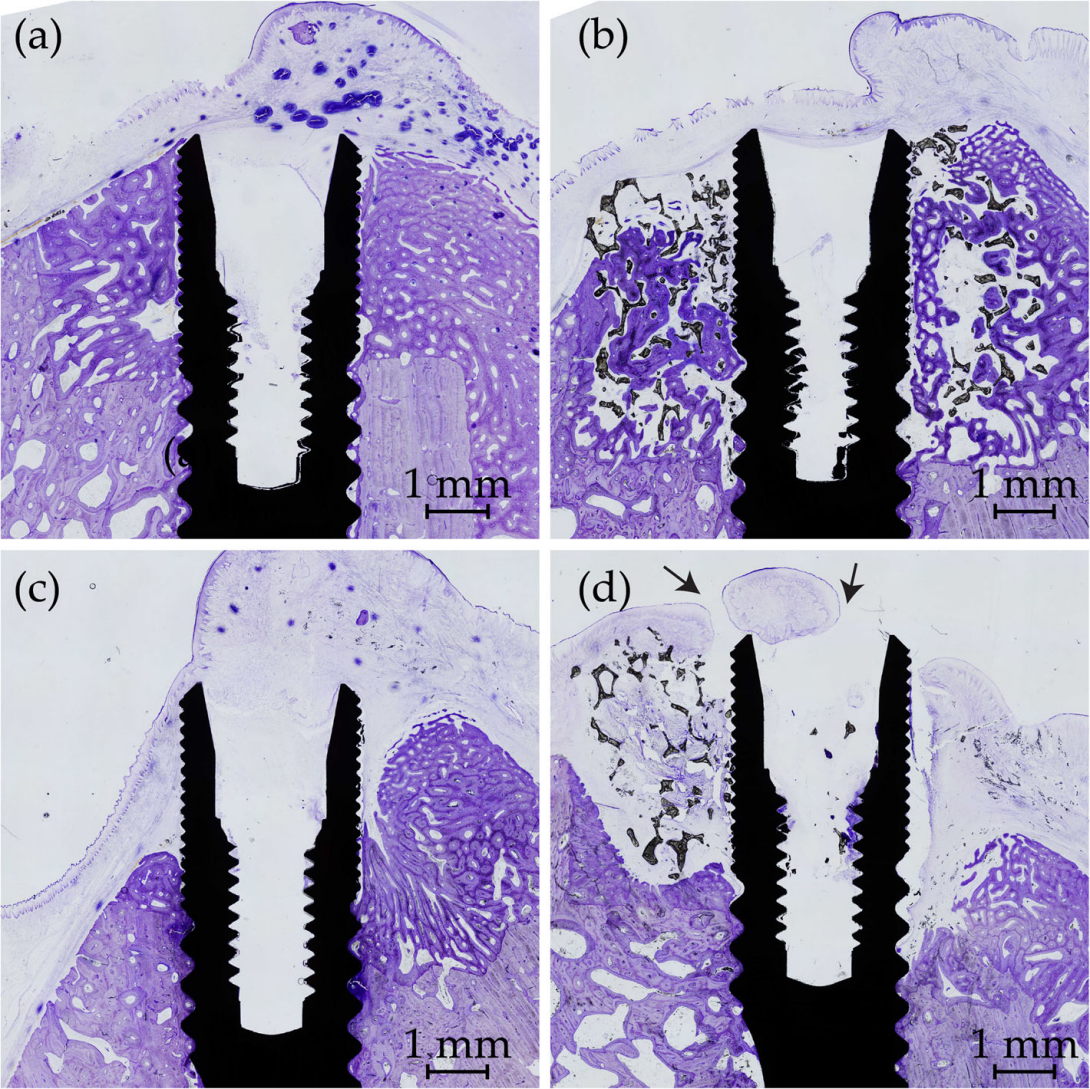
Histological samples of best (**a**-**b**) and worst (**c**-**d**) samples from sham (**a**) and (**c**) and TiO_2_ (**b**) and (**d**). New bone stained deep purple and irregular in shape. Lingual side left and buccal side right for all figures. **a** Complete healing of the defect. **b** Extensive bone growth within the porous scaffold. **c** Resorption of the lingual bone wall and epithelium in contact with the implant. **d** Missing scaffold at the buccal side and no bone contact between bone and scaffold lingually. Arrows: mucosal perforations also visible on both sides

All implants were osseointegrated. New bone formation was clearly distinguishable from the original bone and the defects were readily identified. Except for one sham site (Fig. 5a), no buccal bone wall was completely re-established to the level of the implant shoulder in the defects. The TiO_2_ scaffold which was in part missing on MicroCT showed no contact between the TiO_2_ scaffold and the bone in histology (Fig. 5d). The remaining 11 scaffolds were in intimate contact with the bone and demonstrated new bone formation in various degrees within the porous scaffold structures.

### Defect fill

**Fig. 6:**
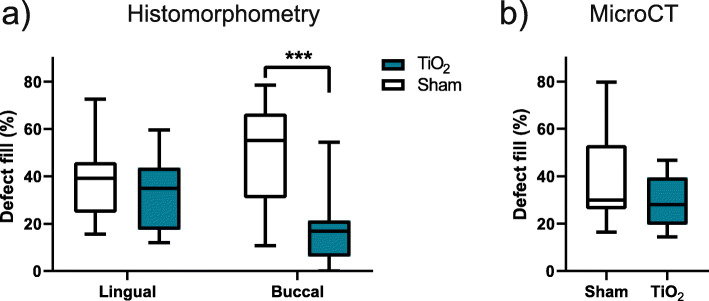
Defect fill measured by (**a**) histomorphometry and (**b**) microCT. *** *p* < 0.001

Results are presented in Fig. 6. Histomorphometric results at the buccal sites demonstrated a statistically significant higher fraction of bone fill between the sham group (median: 55.1%, IQR: 30.8-66.6) compared to the TiO_2_ scaffolds (median: 16.9%, IQR: 6.1-21.3).

### Bone to implant contact

**Fig. 7:**
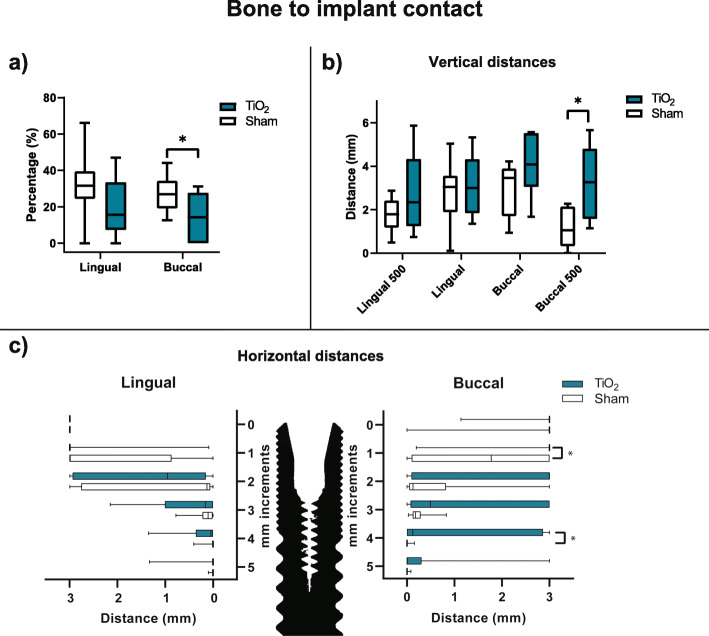
Bone to implant contact by histomorphometry. **a** Percentage of bone to implant contact. **b** First bone to implant contact by vertical measurements at implant surface and 500 μm laterally. **c** First bone to implant contact by horizontal measurements at the upper most 6 mm increments. * *p* < 0.05

The results for %BIC at the uppermost 5 mm of the implants are shown in Fig. 7a. Statistically significant difference was found in the buccal sites between shams (median: 26.9%, IQR: 19.1-34.4) and TiO_2_ blocks (median: 14.3%, IQR: 0.0-27.7). No significant difference was found at the lingual sites.

The results for vertical FBIC and FBIC500 are shown in Fig. 7b. No significant difference in FBIC between TiO_2_ blocks and sham were found in buccal or lingual sites. For FBIC500, the shams (median: 1.1 mm, IQR: 0.3-2.1) demonstrated a significantly lower value on the buccal side than the TiO_2_ blocks (median: 3.3 mm, IQR: 1.6-4.8). No significant difference was found for FBIC500 in lingual sites.

Horizontal width measurements are presented in Fig. 7c. Statistically significant difference was found in buccal sites between sham and TiO_2_ scaffold at millimetre 1 (median: 1.8 mm, IQR: 0.9-3.0 and median: 3.0 mm, IQR: 3.0-3.0, respectively) and millimetre 4 (median: 0 mm, IQR: 0.0-0.0 and median: 1.2 mm, IQR: 0.0-2.9, respectively).

### Soft tissue measurements

**Fig. 8:**
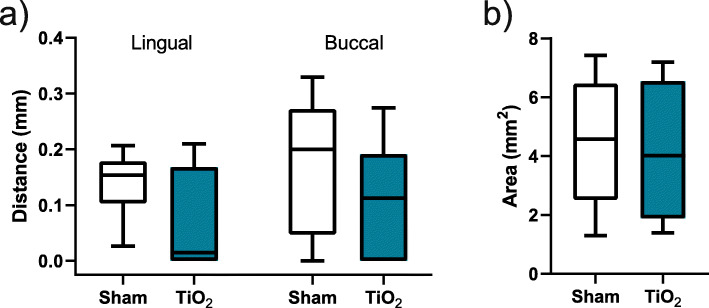
Soft tissue measurements. **a** Shortest soft tissue distance to oral cavity from implant shoulder. **b** Area of soft tissue above the implant

Results are presented in Fig. 8. No statistically significant differences were found between sham and TiO_2_ for either shortest soft tissue distance to oral cavity from implant shoulder or area of soft tissue above the implant.

## Discussion

This study demonstrated less bone formation on the buccal side with the use of TiO_2_ scaffold block along with simultaneous implant placement in circumferential defects with buccal bone fenestrations, as compared to sham sites.

In microCT, no significant difference was seen in comparing %DF. However, the region of interest analysed for %DF was different in histomorphometry and microCT. The microCT analysis set out to evaluate a 3D volume within the scaffolds at the upper part of the implants, excluding the interface between the scaffold and adjacent structures. This volume of interest was chosen to evaluate the space within the porous scaffold structures at the upper part of the implant. As previously demonstrated, bone growth occurs from the defect borders [21]. The 3D microCT analysis did not differentiate between buccal and lingual sites.

The fact that differences in defect fill, bone to implant contact and vertical FBIC500 were observed in the buccal but not lingual sites points to the impact of the lack of a buccal bone wall in this model. The TiO_2_ scaffold did not improve bone healing at this aspect. A similar defect model in dogs by Botticelli et al. demonstrated an incomplete healing when the buccal bone wall was removed [2]. In comparison, they found a defect resolution in four wall defects for both gap distances evaluated (1 and 2.25 mm). This demonstrated buccal bone fenestrations as a challenging model. In contrast to the aforementioned study by Botticelli et al., no collagen membrane was used to cover the defects in the present study. Guided bone regeneration (GBR) is defined as the use of a barrier membrane to direct the growth of new bone and has become a predictable therapeutic method used routinely [22, 23]. Barrier membranes should maintain the space for bone formation and exclude soft tissue invasion of the defect and are commonly used in augmentation procedures. In parallel with its barrier function, the membrane plays an active role in hosting and modulating the molecular activities during GBR [24]. However, their clinical efficacy is debated [25]. Several studies have shown defects healed both with and without membrane for immediate implant placement in four wall defects [26–28]. When used in the bone ring technique, studies have reported no benefits of a membrane [29, 30]. As such, it was opted not to use a membrane in the present study to avoid confounding factors and to assess the bone regeneration capacity of the TiO_2_ scaffolds.

Barrier membranes are also used to ensure the stability of bone graft materials and it can be argued that this aspect is not necessary in a block graft, which furthermore is non-resorbable. Additionally, comparison with the use of empty defects would be cumbersome with the use of membranes as the inadequate mechanical properties of a collagen membrane would likely render insufficient space maintenance in the relatively large defects, compared to the TiO_2_ scaffolds. Closing the site without the use of a membrane covering the scaffold material resulted consequently in a direct contact between the gingival flap and the TiO_2_ scaffold. Soft tissue measurements were performed on histological samples to evaluate any effect of the TiO_2_ scaffold on soft tissue and no differences were found in neither buccal nor lingual sites. The results may indicate that the scaffolds were well tolerated by the soft tissue, even when suspended over the scaffold ridge in the buccal sites. A non-resorbable membrane with enhanced mechanical strength could have been considered and may have had a positive influence on the bone reconstruction and soft tissue dimensions.

In the present study, nine out of twelve TiO_2_ scaffolds were intact at the end of the study. The new bone formation within the porous structures in eleven out of twelve scaffolds may indicate sufficient mechanical stability of the scaffolds for bone healing. Fractured scaffold edges were not found as previously reported [31]. This can be explained by three-wall defects in the present study, which provide stability and shelters the scaffold from external forces in contrast to its use in lateral bone augmentation models. The aforementioned study also used a fixation screw to secure the block, which resulted in partial scaffold fracture around the screw due to stress concentration. The press-fit installation of the scaffold may have provided more even stress distribution for the brittle TiO_2_ scaffolds, thereby maintained the augmented volume throughout the evaluated period. In both studies, the scaffold allowed easy chairside adaptation with dental burs or scissors. This enabled efficient shaping of the scaffold to fill the augmented space, which is advantageous for bone graft substitute materials [10]. In comparison, particulate graft materials often require a flexible membrane for mechanical stabilisation, which also limits the expandable space. Alternatively, a stiff barrier like titanium meshes needs to be manually bent and shaped in a time consuming process and ill adapted meshes increase the risk of mucosal rupture and exposure of the mesh [32].

Mucosal perforations to the oral cavity were found clinically at five sites, four of which in the TiO_2_ scaffold group. An implant cover screw, which is temporarily used to close the implant lumen during osseointegration, could have potentially resulted in less mucosal perforations. However, bone growth was confirmed within the porous scaffold structures at all but one of the TiO_2_ scaffold sites. As it was a challenging defect model, a longer healing time might show continued bone growth, and the single time point evaluated is a limiting factor of this study. In comparison, when GBR is performed in a two staged approach, implant placement is carried out from 6 to 12 months after GBR surgery [33].

The results suggest that the TiO_2_ scaffolds hold the basic requirements for a bone substitute; osteoconductive, biocompatible, space-making capability and volume maintenance [34], also confirmed in previous in vivo studies [18, 19, 31]. However, the TiO_2_ scaffolds are lacking osteoinductive properties as bone formation is seen only from the lateral borders of the grafts, originating from the parent bone. Different strategies have been proposed to improve new bone formation and in vitro studies applying various coatings to the scaffold have shown possibilities for increased osteogenic potential [35–37]. More recently, cationic doping of TiO_2_ scaffolds with Ca, Sr or Mg have shown ion release in addition to the benefit of increased mechanical strength [38]. The released Mg resulted in significantly increased osteogenic differentiation. As the ions Ca, Sr and Mg influence bone biology [39–41], doping could be a prospect for further investigation.

## Conclusions

Less bone formation was observed on the buccal side with the use of TiO_2_ scaffold block in combination with simultaneous implant placement in circumferential defects with buccal bone dehiscences, as compared to sham sites.

## Data Availability

All data is available upon request to the corresponding author.

## Abbreviations

%BIC: Percentage of bone-to-implant contact
FBIC: First bone-to-implant contact
FBIC500: First bone-to-implant contact 500 μm lateral to the implant
GBR: Guided bone regeneration
MicroCT: Microcomputed tomography
TiO_2_: Titanium dioxide
VOI: Volume of interest

## References

[1] Schropp L, Wenzel A, Kostopoulos L, Karring T (2003). Bone healing and soft tissue contour changes following single-tooth extraction: a clinical and radiographic 12-month prospective study. Int J Periodontics Restorative Dentistry.

[2] Botticelli D, Berglundh T, Lindhe J (2004). Resolution of bone defects of varying dimension and configuration in the marginal portion of the peri-implant bone. J Clin Periodontol.

[3] Paolantonio M, Dolci M, Scarano A, D'Archivio D, Di Placido G, Tumini V (2001). Immediate implantation in fresh extraction sockets. A controlled clinical and histological study in Man. J Periodontol.

[4] Ortega-Martínez J, Pérez-Pascual T, Mareque-Bueno S, Hernández-Alfaro F, Ferrés-Padró E (2012). Immediate implants following tooth extraction. A systematic review. Med Oral Patol Oral Cir Bucal.

[5] Nkenke E, Schultze-Mosgau S, Kloss F, Neukam FW, Radespiel-Tröger M (2001). Morbidity of harvesting of chin grafts: a prospective study. Clin Oral Implants Res.

[6] Nkenke E, Neukam FW (2014). Autogenous bone harvesting and grafting in advanced jaw resorption: morbidity, resorption and implant survival. Eur J Oral Implantol.

[7] Araújo MG, Lindhe J (2011). Socket grafting with the use of autologous bone: an experimental study in the dog. Clin Oral Implants Res.

[8] Sanz-Sánchez I, Ortiz-Vigón A, Sanz-Martín I, Figuero E, Sanz M (2015). Effectiveness of lateral bone augmentation on the alveolar crest dimension: a systematic review and Meta-analysis. J Dent Res.

[9] Sogal A, Tofe AJ (1999). Risk assessment of bovine spongiform encephalopathy transmission through bone graft material derived from bovine bone used for dental applications. J Periodontol.

[10] Haugen HJ, Lyngstadaas SP, Rossi F, Perale G (2019). Bone grafts: which is the ideal biomaterial?. J Clin Periodontol.

[11] Giesenhagen B, Martin N, Donkiewicz P, Perić Kačarević Ž, Smeets R, Jung O (2018). Vertical bone augmentation in a single-tooth gap with an allogenic bone ring: clinical considerations. J Esthet Restor Dent.

[12] Miller RJ, Korn RJ, Miller RJ (2020). Indications for simultaneous implantation and bone augmentation using the allograft bone ring technique. Int J Perio Res Dent.

[13] Sáez-Alcaide LM, Brinkmann JC-B, Sánchez-Labrador L, Pérez-González F, Molinero-Mourelle P, López-Quiles J (2020). Effectiveness of the bone ring technique and simultaneous implant placement for vertical ridge augmentation: a systematic review. Int. J Implant Dent.

[14] Gaikwad AM, Joshi AA, Padhye AM, Nadgere JB. Autogenous bone ring for vertical bone augmentation procedure with simultaneous implant placement: a systematic review of histologic and histomorphometric outcomes in animal studies. J Prosthet Dent. 2021;126(5):626–35. 10.1016/j.prosdent.2020.09.001. 10.1016/j.prosdent.2020.09.001 33039188

[15] Sabetrasekh R, Tiainen H, Lyngstadaas SP, Reseland J, Haugen H (2010). A novel ultra-porous titanium dioxide ceramic with excellent biocompatibility. J Biomater Appl.

[16] Tiainen H, Wiedmer D, Haugen HJ (2013). Processing of highly porous TiO2 bone scaffolds with improved compressive strength. J Eur Ceram Soc.

[17] Tiainen H, Lyngstadaas SP, Ellingsen JE, Haugen HJ (2010). Ultra-porous titanium oxide scaffold with high compressive strength. J Mater Sci Mater Med.

[18] Tiainen H, Wohlfahrt JC, Verket A, Lyngstadaas SP, Haugen HJ (2012). Bone formation in TiO2 bone scaffolds in extraction sockets of minipigs. Acta Biomater.

[19] Verket A, Müller B, Wohlfahrt JC, Lyngstadaas SP, Ellingsen JE, Jostein Haugen H (2016). TiO2 scaffolds in peri-implant dehiscence defects: an experimental pilot study. Clin Oral Implants Res.

[20] Botticelli D, Berglundh T, Lindhe J (2004). The influence of a biomaterial on the closure of a marginal hard tissue defect adjacent to implants. Clin Oral Implants Res.

[21] Botticelli D, Berglundh T, Buser D, Lindhe J (2003). Appositional bone formation in marginal defects at implants. Clin Oral Implants Res.

[22] Hämmerle CH, Karring T (1998). Guided bone regeneration at oral implant sites. Periodontology 2000.

[23] Buser D, Dula K, Belser UC, Hirt H-P, Berthold H. Localized ridge augmentation using guided bone regeneration. II. Surgical procedure in the mandible. Int J Periodontics Restorative Dentistry. 1995;15(1):10–29. 7591520

[24] Omar O, Elgali I, Dahlin C, Thomsen P (2019). Barrier membranes: more than the barrier effect?. J Clin Periodontol.

[25] Hu C, Gong T, Lin W, Yuan Q, Man Y (2017). Immediate implant placement into posterior sockets with or without buccal bone dehiscence defects: a retrospective cohort study. J Dent.

[26] Chen ST, Darby IB, Reynolds EC (2007). A prospective clinical study of non-submerged immediate implants: clinical outcomes and esthetic results. Clin Oral Implants Res.

[27] Covani U, Cornelini R, Barone A (2003). Bucco-lingual bone remodeling around implants placed into immediate extraction sockets: a case series. J Periodontol.

[28] Botticelli D, Berglundh T, Buser D, Lindhe J (2003). The jumping distance revisited. Clin Oral Implants Res.

[29] Haga-Tsujimura M, Nakahara K, Kobayashi E, Igarashi K, Schaller B, Saulacic N (2018). Single-staged implant placement using bone ring technique with and without membrane placement: an experimental study in the beagle dog. Clin Oral Implants Res.

[30] Nakahara K, Haga-Tsujimura M, Igarashi K, Kobayashi E, Schaller B, Lang NP (2020). Single-staged implant placement using the bone ring technique with and without membrane placement: Micro-CT analysis in a preclinical in vivo study. Clin Oral Implants Res.

[31] Thieu MKL, Haugen HJ, Sanz-Esporrin J, Sanz M, Lyngstadaas SP, Verket A (2021). Guided bone regeneration of chronic non-contained bone defects using a volume stable porous block TiO2 scaffold: an experimental in vivo study. Clin Oral Implants Res.

[32] Xie Y, Li S, Zhang T, Wang C, Cai X (2020). Titanium mesh for bone augmentation in oral implantology: current application and progress. Int J Oral Sci.

[33] Buser D, Chappuis V, Belser UC, Chen S (2017). Implant placement post extraction in esthetic single tooth sites: when immediate, when early, when late?. Periodontology 2000.

[34] Yamada M, Egusa H (2018). Current bone substitutes for implant dentistry. J Prosthodont Res.

[35] Verket A, Tiainen H, Haugen HJ, Lyngstadaas SP, Nilsen O, Reseland JE (2012). Enhanced osteoblast differentiation on scaffolds coated with TiO2 compared to SiO2 and CaP coatings. Biointerphases..

[36] Pullisaar H, Reseland JE, Haugen HJ, Brinchmann JE, Ostrup E (2014). Simvastatin coating of TiO_2_ scaffold induces osteogenic differentiation of human adipose tissue-derived mesenchymal stem cells. Biochem Biophys Res Commun.

[37] Rubert M, Pullisaar H, Gómez-Florit M, Ramis JM, Tiainen H, Haugen HJ (2013). Effect of TiO2 scaffolds coated with alginate hydrogel containing a proline-rich peptide on osteoblast growth and differentiation in vitro. J Biomed Mater Res A.

[38] Klemm A, Gomez-Florit M, Carvalho PA, Wachendörfer M, Gomes ME, Haugen HJ (2019). Grain boundary corrosion in TiO2 bone scaffolds doped with group II cations. J Eur Ceram Soc.

[39] Yoshizawa S, Brown A, Barchowsky A, Sfeir C (2014). Magnesium ion stimulation of bone marrow stromal cells enhances osteogenic activity, simulating the effect of magnesium alloy degradation. Acta Biomater.

[40] Pors NS (2004). The biological role of strontium. Bone..

[41] Blair HC, Robinson LJ, Huang CL-H, Sun L, Friedman PA, Schlesinger PH (2011). Calcium and bone disease. BioFactors..

